# Self-reported sleep and exercise patterns in patients admitted with suicidal attempts: a cross-sectional comparative study

**DOI:** 10.1186/s12888-022-03929-9

**Published:** 2022-05-09

**Authors:** Manaal Siddiqui, Hassen Al-Amin, Mahmoud Abu Rabeh, Mahmoud Meedany, Yasmin Hamdi, Suhaila Ghuloum

**Affiliations:** 1 Qatar Foundation, Weill Cornell Medicine-Qatar, Education City, 24144 Doha, Qatar; 2 Department of Psychiatry, Hamad Medical Corporation, Doha, Qatar

**Keywords:** Sleep, Exercise, Suicide, Insomnia

## Abstract

**Background:**

There is evidence that sleep disturbances and exercise are risk factors for suicide attempts; however, whether sleep disturbances are independently associated with suicide attempts is debatable. We compared the sleep and exercise patterns of individuals who attempted suicide to those of the general population and investigated whether sleep disturbances were independently associated with suicide attempts.

**Methods:**

Over a year, individuals presented to the emergency department at Hamad General Hospital and Mental Health Services in Doha with suicide attempts (*n* = 127) filled out questionnaires on sleep and exercise, demographics, and clinical measures. A control group (*n* = 126) from two primary care centers filled out the same questionnaires during the same period.

**Results:**

Subjects in the suicide group were significantly younger, single, had a lower level of education, and showed considerably more early insomnia, daytime tiredness, interrupted sleep, and no regular exercise. The most common diagnoses seen with suicidality were adjustment disorder and major depression, and the most common method used to attempt suicide was an overdose. After multiple regression analysis, being Arab, belonging to the category “other nationalities,” unemployment, and early insomnia were significantly associated with an increased risk of suicide attempts.

**Conclusion:**

This is the first comparative study on suicide in the Arabian Gulf. Individuals in Qatar with acute stress, depressive symptoms, sleep disturbances, and lack of exercise are at increased risk of attempting suicide. Thus, clinicians need to routinely screen for sleep and physical activity because of their significant contribution to physical and mental well-being.

## Introduction

Every year, approximately 800,000 people die by suicide [1], and even more suffer from suicidal thoughts and behaviors [2]. Thus, there is an increasing need to identify the risk factors and warning signs for suicide to achieve timely interventions and minimize the global burden of suicidality. Sleep disturbances are also a significant public health concern, interfering with daily functioning, and negatively impacting mental and physical health. According to several cross-sectional and population-based studies conducted in various clinical populations, including adults, adolescents, war veterans, and children, sleep disturbance is a putative risk factor and an acute warning sign for suicidal thoughts and behaviors [3–6]. However, it is debatable whether this association is dependent on depression, anxiety, post-traumatic stress symptoms, and other psychiatric illnesses [2]. According to a 2015 systematic review and a 2012 meta-analysis, sleep disturbances were independently associated with an elevated risk of suicide attempts [7, 8], and another recent prospective study reached the same conclusion [9].

Furthermore, after a posteriori analysis of existing evidence, two recent systematic reviews concluded that sleep disturbances could be a potential biomarker for suicidal behavior [10, 11]. Another report showed that 92% of subjects with severe suicidal attempts had difficulty falling asleep, interrupted sleep, or early morning awakening [12]. In contrast, others reported the loss of this significance after controlling for confounding variables such as depression, anxiety, and post-traumatic stress symptoms [2, 13]. Thus, sleep disturbances increase the long-term and short-term risk of suicidal thoughts and behaviors, but whether this association is related to other clinical and demographic factors has yet to be elucidated.

Effective suicide prevention programs need to focus on modifiable measures, like sleep and exercise, for individuals at risk of suicide. The latter is known to offer resilience in the face of life’s stressors and confer a protective effect on mental health and suicide attempts [14]. Exercise has shown efficacy in treating depression with effect sizes similar to those of medications [15]. It has also led to increased physical and psychological well-being in patients with depression [16]. It also exhibits a mood-stabilizing effect in bipolar disorder in adjunct to medications. Exercise may mediate these effects by increasing brain-derived nerve factor (BDNF), decreasing oxidative stress, and inducing epigenetic changes beneficial for mood disorders [15]. Furthermore, aerobic exercise is also known to enhance the ability to regulate emotions through increasing executive function. In a randomized control trial, acute aerobic exercise increased the capacity to downregulate negative emotions in response to unpleasant emotional cues [17]. Regarding suicide attempts, a case-controlled study showed an independent protective association between physical activity and the risk of suicide attempts [18]. An extensive web-based survey among Korean adolescents showed that those who met the recommended guidelines for physical activity were less likely to report past suicide attempts [19]. Similarly, in a population-based, case-controlled study, Simon et al. found that those who attempted suicide were less likely to report involvement in physical activity in the past month than controls [18].

There are also ethnic and cultural variations in these risk and protective factors [20] and their subsequent impact on suicidal thoughts and behaviors. For instance, there are geographical differences in sleep patterns [21], and the prevalence of insufficient physical activity also varies across countries [22]. Thus, it is crucial to investigate the risk factors for suicide in various settings across the globe. For example, Qatar is one of the Islamic countries in the Arab peninsula known to have a low suicide rate [23]. These apparent low rates are due to a lack of national suicide registries and the stigma surrounding suicide, an act condemned by Islam and considered sinful. This denunciation prompts victims and their families to conceal self-harm behaviors and suicide intents [24]. So, in regions where individuals are disinclined to report their mental health symptoms, better screening techniques based on the risk factors may early identify those at an increased risk for attempting suicide.

The present study evaluated the risk factors and warning signs for suicide attempts in Qatar. In addition, this study assessed the sleep and exercise patterns in patients with suicide attempts compared to a general population sample with no psychiatric illness or suicide attempts. We hypothesized that the group with suicide attempts would have more sleep problems and less exercise activity than controls. Moreover, we investigated whether sleep disturbances were associated with an increased risk of suicide attempts and whether this association occurred independently of other socio-demographical and clinical characteristics.

## Methods

This is a cross-sectional, case-controlled study assessing the relationship between sleep and suicide in Qatar. We conducted the study between January 2015 and January 2016, and the Institutional Review Boards (IRBs) at both Hamad Medical Corporation (HMC) and Weill Cornell Medicine (WCM-Q) in Doha, Qatar, approved the study. All subjects signed a written consent after explaining the study’s purpose, procedures, and duration.

### Setting and participants

Qatar is a rapidly growing country with a strong economy. The population of Qatar at the time of the study was about 2,400,000 [25], where the majority were expatriates (over 80%) from South Asia [26]. The Mental Health Services is the only psychiatric facility in Doha with 80 beds inpatient capacity (95–100% occupancy) and ten outpatient clinics with daily visits for about 100 patients per weekday. We recruited the subjects with suicidal attempts (*n* = 127) through the Emergency Department of HMC and Mental Health Services in Doha during the one year indicated above. Another 35 subjects were approached to participate, but they refused. We enlisted the control group (*n* = 126) from two primary care centers in Doha and the Mental Health Services' visitors during the same period. Another 43 subjects were approached for the control group, but they refused. The inclusion criteria for the suicide group were: age 18–65 years, recent suicidal attempt within the last week, and the ability to sign written informed consent (English or Arabic). We established the suicide risk and intent using the Columbia-Suicide Severity Rating Scale (C-SSRS) [27]. The exclusion criteria were current medical problems that require immediate medical attention. The control group had the same criteria of age, absence of ongoing medical issues, and the capacity to sign consent. Also, they did not have any history of psychiatric disease or suicidal attempts.

### Procedure and measurements

All the subjects answered questionnaires to cover the sociodemographics, medical and psychiatry histories, sleep, and exercise. The questionnaires and scales were available in both English and Arabic. The sleep questionnaire is a modified version of the Pittsburgh Sleep Quality Index (PSQI) [28], and the research team designed the exercise questionnaire. The Arabic versions (translated by bilingual clinicians) were culturally adapted for Arabic-speaking populations [29, 30]. These instruments were piloted in Arabic and English-speaking patients and controls in Qatar, and their feedback was reflected in the final versions. We instructed the participants to answer the sleep and exercise questions based on their experiences during the month before the suicidal attempt. The research team trained the bilingual raters to administer these questionnaires before the start of the study. They were randomly assigned on different days to administer the questionnaires to both the suicide and control groups.

### Statistical analysis

We analyzed the data using the IBM SPSS version 26 (IBM Corp, Armonk, NY, 2015), with the significance level set at 0.05. We present the continuous variables as mean ± standard deviation (S.D.) and the categorical ones as frequency distributions. We compared the two groups using independent samples t-test for continuous variables, chi-square test for categorical ones, and Bonferroni corrections (option in SPSS) for multiple comparisons. The Kolmogorov–Smirnov test was used to assess normality. In this regard, we used Mann–Whitney U. We wanted to evaluate if sleep problems are independently associated with suicide. We used multiple logistic regression (backward conditional) using the significant variables from the bivariate analysis (control vs. suicide group) and the clinically relevant factors from other studies. We tested the best-fit model using Hosmer and Lemeshow test and by checking Nagelkerke R^2^.

## Results

### ***Sociodemographic characteristics of the sample (Table ******1******)***

**Table 1:** Sociodemographic characteristics of study participants

	**Control group** (*n* = 126)	**Suicide group** (*n* = 127)
**Age** *(mean* ± *SD)*	35.05 ± 8.17^ab^	29.55 ± 7.30
**Age groups** *n (%)*		
≤ 24 years	7 (5.6%)	28 (22.0%)^a^
25–34 years	62 (49.2%)	73 (57.5%)
35–44 years	39 (31.0%)^ab^	20 (15.7%)
≥ 45 years	18 (14.3%)^ab^	6 (4.7%)
**Gender** *n (%)*		
Male	72 (57.1%)	64 (50.4%)
Female	54 (42.9%)	63 (49.6%)
**Nationality** *n (%)*		
Qatari	22 (17.5%)	16 (12.6%)
Arab (non-Qatari)	35 (27.8%)	31 (24.4%)
South Asian	57 (45.2%)	43 (33.9%)
Other	12 (9.5%)	37 (29.1%)^a^
**Marital status** *n (%)*		
Single	35 (27.8%)	64 (41.7%)^a^
Married	90 (71.4%)^ab^	53 (77.2%)
Divorced/Widowed	1 (0.8%)	10 (7.9%)^a^
**Number of children**		
No children	54 (42.9%)	73 (57.5%)
1–2	39 (31.0%)	32 (25.2%)
3 and more	33 (26.2%)	22 (17.3%)
**Education** *n (%)*		
No schooling	5 (4.0%)	7 (5.5%)
Elementary/Intermediate	20 (15.9%)	45 (35.4%)^a^
Secondary/Vocational	45 (35.7%)	46 (36.2%)
College/Postgraduate	56 (44.4%)^ab^	29 (22.8%)
**Employment** *n (%)*		
Employed	116 (92.1%)^ab^	82 (64.6%)
Unemployed	10 (7.9%)	45 (35.4)^a^

^a^higher than the control group; ^ab^higher than the suicide group (*p* < 0.05)

Arabs immigrants were from Bahrain, Egypt, Jordan, Lebanon, Morocco, Palestine, Saudi Arabia, Sudan, Syria, Tunisia, and Yemen. South Asians were from Bangladesh, India, Pakistan, Sri Lanka, and Nepal. Other immigrants were from Philippines, Indonesia, Iran, Thailand, Indonesia, Canada, Ethiopia, France, Kenya, Jamaica, Nigeria, South Africa, Tanzania, Germany, United Kingdom, United States of America, and Slovakia

There were no significant gender differences between the suicide cases and controls. The mean age of the suicide group was significantly less than the one for the control group (Table 1). The factors that were significant included marital status (χ^2^ = 25.43, *p* < 0.001), nationality (χ^2^ = 15.90, *p* < 0.001), education degree (χ^2^ = 18.53, *p* < 0.001), and employment status (χ^2^ = 28.11, *p* < 0.001). The suicide group had significantly more single or divorced/widowed subjects than the control group (*p* < 0.05). There were more subjects in the suicide group whose nationality was among the category “Other.” The latter included mainly the Philippines and a few from France, the United Kingdom, and the United States (Table 1). There was a trend for the factor related to the number of children, but it did not reach significance (*p* = 0.057), where the suicide group had a larger number of subjects with no children compared to the control group. The number of subjects with college/postgraduate education and the employed ones was significantly higher in the control group compared to those with suicidal attempts (*p* < 0.05) (Table 1).

### Psychiatric profile of the suicide group (Table 2)

**Table 2:** Psychiatric profile of suicide group

**Variables**	**Suicide group** (*n* = 127)
**Past Psychiatric diagnosis** *n (%)*	
Depression	13 (10.2%)
Bipolar disorder	3 (2.4%)
Anxiety disorder	1 (0.8%)
Eating disorder	1 (0.8%)
Psychotic disorder	1 (0.8%)
Personality disorder	1 (0.8%)
Substance use	2 (1.6%)
“Do not know.”	12 (9.5%)
None	93 (73.2%)
**Hospital Psychiatric diagnosis** *n (%)*	
Depression	33 (26.0%)
Bipolar disorder	4 (3.2%)
Anxiety disorder	1 (0.8%)
Adjustment disorder	40 (31.5%)
Psychotic disorder	10 (7.8%)
Personality disorder	4 (3.2%)
Substance use	13 (10.2%)
Suicide attempt	18 (14.2%)
More than one diagnosis	4 (3.2%)
**Mean of Attempt Categorized**	
Poison	22 (17.5%)
Overdose	50 (39.7%)
Cut/Stab	32 (25.4%)
Hanging	10 (7.9%)
Jumping off Building	4 (3.2%)
Jumping in front of Car	1 (0.8%)
Drowning	2 (1.6%)
Electrocution	1 (0.8%)
Banging Head against the wall	1 (0.8%)
More than one of the above	3 (2.4%)
**Past psychiatric hospitalizations** *n (%)*	26 (20.5%)
**History of suicide attempts** *n (%)*	33 (26.0%)
**Family history/suicide attempt**	5 (3.9%)
**History of aggressive behavior** *n (%)*	17 (13.4%)
**Substance Use**	
Smoking	43 (33.9%)
Alcohol	7 (5.5%)
THC	6 (4.7%)
Opioids	1 (0.8%)
Benzodiazepines	2 (1.6%)
Other	3 (2.4%)

Table 2 summarizes the psychiatric features of the suicide group. Only 26.8% of the subjects who attempted suicide had past psychiatric diagnoses, where the majority had depression. During hospitalization, the majority received the diagnosis of adjustment disorder (31.5%), followed by major depression episode (26.0%), suicidal attempt (14.2%), and substance use disorder (10.2%). The most common method used to attempt suicide was an overdose (39.7%), followed by cutting wrists or stabbing themselves (25.4%), and then consuming poisonous material (17.5%). About 20.5% were hospitalized to a psychiatric hospital before, and 26.0% had a previous suicide attempt. The most common substance used was nicotine (smoking cigarettes) in 33.9% of the sample.

### ***Sleep patterns in suicide and control groups (Table ******3******)***

**Table 3:** Sleeping patterns of study participants

	**Control group** (*n* = 126)	**Suicide group** (*n* = 127)
**Sleep hours** *n (%)*		
Less than 6 h	53 (42.1%)	50 (39.4%)
6–7 h	43 (34.1%)^ab^	23 (18.1%)
7–8 h	23 (18.3%)	25 (19.7%)
8–9 h	6 (4.8%)	16 (12.6%)^a^
More than 9 h	1 (0.8%)	13 (10.2%)^a^
**Time of sleep** *n (%)*		
Between 7 and 9 pm	5 (4.0%)	15 (12.1%)^a^
Between 9 and 11 pm	41 (32.5%)	48 (38.7%)
Between 11 pm and 1 am	67 (53.2%)^ab^	30 (24.2%)
Between 1 and 3 am	10 (7.9%)	14 (11.3%)
After 3 am	3 (2.4%)	17 (13.7%)^a^
**Time of wakeup** *n (%)*		
Between 2 and 4 am	10 (7.9%)	7 (5.5%)
Between 4 and 6 am	86 (68.3%)^ab^	48 (37.8%)
Between 6 and 8 am	24 (19.0%)	41 (32.3%)^a^
Between 8 and 10 am	3 (2.4%)	10 (7.9%)^a^
After 10 am	3 (2.4%)	21 (16.5%)^a^
**Take day naps** *n (%)*	51 (40.5%)	53 (41.7%)
Less than 1 h	15 (11.9%)	15 (11.8%)
Between 1 and 2 h	26 (20.6%)	29 (22.8%)
Between 2 and 3 h	8 (6.3%)	8 (6.3%)
Between 3 and 4 h	2 (1.6%)	1 (0.8%)
**Difficulty falling asleep** *n (%)*	20 (15.9%)	46 (36.2%)^a^
**Early wakeup** *n (%)*	31 (24.6%)	41 (32.3%)
**Interrupted sleep** *n (%)*	21 (16.7%)	41 (32.3%)^a^
**Day tiredness/sleepiness** *n (%)*	37 (29.4%)	56 (44.1%^a^
**Problems during sleep** *n (%)*		
Snoring	25 (19.8%)^ab^	6 (4.7%)
Sleep apnea	2 (1.6%)	2 (1.6%)
**Self-reported sleep quality** *n (%)*		
1 Worst	4 (3.2%)	8 (6.9%)
2	6 (4.8%)	23 (19.8%)^a^
3	27 (21.4%)	31 (26.7%)
4	46 (36.5%)	31 (26.7%)
5 Best	43 (34.1%)^ab^	23 (19.8%)

^a^higher than the control group; ^ab^ higher than the suicide group (*p* < .05)

All the sleep measures were significantly different between the two groups except for day naps and early wake up (Table 3): sleep hours (χ^2^ = 21.06, *p* < 0.001), wakeup (χ^2^ = 33.02, *p* < 0.001) and sleep (χ^2^ = 30.12, *p* < 0.001) times, early insomnia (χ^2^ = 13.58, *p* < 0.001), interrupted sleep (χ^2^ = 8.34, *p* < 0.004), day tiredness/sleepiness (χ^2^ = 5.90, *p* < 0.015), sleep problems (χ^2^ = 25.43, *p* < 0.001), and sleep quality (χ^2^ = 20.18, *p* < 0.001). Comparisons revealed an equal number of subjects in each group sleeping for less than six hours. More subjects in the control group slept 6–7 h, and more subjects in the suicide group slept more than 8 h (*p* < 0.05). The suicide group showed more subjects who would sleep either early or late (and woke up later in the morning) while most of the control group slept between 11 pm and 1 am (and woke up between 4 and 6 am) (Table 1).

The suicide group showed significantly more subjects with early insomnia, interrupted sleep, and day tiredness than the control group. The sleep problems were not common in the suicide group, and the number of subjects with snoring was higher in the control group. More subjects in the control group scored excellent sleep quality (higher values mean better quality) compared to the suicide one (p < 0.05) (Table 3). We also analyzed the gender differences in the sleep patterns in the two groups (suicide vs. controls). Many of the sleep disturbances were significantly higher in the males attempting suicide than controls (early insomnia, χ^2^ = 19.66, *p* < 0.001; late insomnia, χ^2^ = 12.06, *p* = 0.001; interrupted sleep, χ^2^ = 21.75, *p* < 0.001) but not in the females. The sleep duration (χ^2^ = 18.97, *p* = 0.001), mainly sleeping less than six hours, and poor sleep quality (score < 3) (χ^2^ = 31.75, *p* < 0.001) were also more affected in the males of the suicide group (compared to controls) but not in the female ones.

### Exercise patterns in suicide and control groups (Table 4)

**Table 4:** Exercise patterns of study participants

	**Control group** (*n* = 126)	**Suicide group** (*n* = 127)
**Frequency of exercise** *n (%)*		
Once per week	27 (21.4%)^ab^	9 (7.1%)
2–4 times per week	22 (17.5%)	17 (13.4%)
> 5 times per week	15 (11.9%)	10 (7.9%)
Never	62 (49.2%)	91 (71.7%)^a^
**Duration of exercise** *n (%)*		
< 15 min	7 (5.6%%)	3 (2.4%)
15–30 min	31 (24.6%)^ab^	11 (8.7%)
30–60 min	19 (15.1%)	14 (11.0%)
> 1 h	7 (5.6%)	8 (6.3%)
**Intensity of exercise** *n (%)*		
Light	41 (32.5%)^ab^	16 (12.6%)
Moderate	15 (11.9%)	9 (7.1%)
High	8 (6.3%)	11 (8.7%)
**Reason for exercise** *n (%)*		
Reducing/maintaining weight	16 (12.7%)^ab^	8 (6.3%) 19
General health	35 (27.8%)^ab^	(15.0%)
Pleasure/leisure	4 (3.2%)	14 (11.0%)^a^
Stress relief	1 (0.8%)	2 (1.6%)
Other	7 (5.6%)^ab^	1 (0.8%)

^a^higher than the control group; ^ab^higher than the suicide group (*p* < .05)

All the variables on exercise were significantly different between the two groups: frequency (χ^2^ = 16.13, *p* = 0.001), duration (χ^2^ = 17.44, *p* = 0.002), and intensity (χ^2^ = 18.43, *p* < 0.001) (Table 3). The majority of the suicide group (71.7%) did not exercise at all. Among those who exercised, most of them were doing light exercise, 15–30 min one time per week, and these were mainly in the control group to manage weight and for general well-being (Table 1).

### Multiple regression analysis

We conducted multiple regression analyses to check if sleep problems are independently associated with suicide after controlling for the other factors contributing to suicidality and sleep problems. The dependent factor was the group (control vs. suicide). The independent factors were age, gender, marital status, employment, having children, nationality, frequency of exercise, early insomnia, interrupted sleep, and late insomnia. The regression model with all the predictors was significant (χ^2^ = 144.98, df = 14, and *p* < 0.001). The Nagelkerke R^2^ showed that the model explained 58.2% of the variance predicted by these variables. The best-fit model was confirmed by the Hosmer and Lemeshow test (χ^2^ = 2.09, df = 8, and *p* = 0.98). The model gave an overall 79.4% correct rate of the outcome (control vs. suicide). The predictors that remained significantly associated with suicide after controlling for the other ones were nationality, employment, and having early insomnia (Table 5). Compared to Qataris, Arabs and other nationalities are 4.14 and 15.79 times, respectively, more likely to attempt suicide. The odds of unemployed subjects attempting suicide are 4.8 times more than the employed ones. Those with early insomnia were 2.58 times more likely to attempt suicide than those without such a sleep problem.

**Table 5:** Predictors of suicidality using multiple logistic regression

Independent contributors	Exp (B)	95% CI for Exp (B)		*p*-value
		**Lower Bound**	**Upper Bound**	
**Nationality** (Ref. Qataris)				
Arabs	4.15	1.19	12.27	0.02
South Asia	2.92	0.81	8.09	0.11
Other	15.79	4.09	60.98	< 0.001
**No employment **(Ref. employed)	4.80	1.78	12.96	0.002
**Early insomnia **(Ref. No early insomnia)	2.58	1.03	6.49	0.04

*Ref* reference category

## Discussion

This study compared the sociodemographic characteristics and the sleep/exercise patterns between subjects with suicide attempts and those from the general population with no psychiatric disorders or suicide attempts. Additionally, we investigated whether sleep disturbances were independently associated with an increased risk of suicide attempts. We found that subjects with early insomnia, being expatriate, and unemployed, were independently associated with an increased risk of attempting suicide after controlling for age, gender, and other sociodemographic factors. Furthermore, those in the suicide group were less likely to report involvement in physical activity than individuals in the control group. We will discuss our results focusing on the aims mentioned above.

### Sample characteristics and suicide

In the population residing in Qatar, the following features were significantly different in the suicide group when compared to the control one: young age (≤ 24 years), expatriate (other than south Asians), single, divorced/widowed, unemployed, lower education status, and unemployment (Table 1). This profile is similar to the previously reported risk factors for suicidality in other Arab countries [31], the Middle East North Africa region [32], and around the world [33–35]. In addition, females of a young age are at risk of attempting suicide in most populations [33, 36–38]. However, the 1:1 male to female in our suicide group (Table 1) does not follow the known pattern where females tend to attempt suicide more than males. This discrepancy can be explained by the fact that the male to female ratio in the population of Qatar is about 3 [39]. Thus, the ratio in our sample does not reflect the ratio of the population in Qatar but indirectly indicates that there were more females attempting suicide in this population.

Our study showed that expatriates overall are more predisposed to attempt suicide than nationals in Qatar. Other neighboring countries in the Gulf Cooperation Council, where a high proportion of the population is expatriates, also showed the same trend. For example, Saudi Arabia, Kuwait, and Bahrain reported a higher percentage of deaths by suicide in the expatriate populations than in the local community [40–42]. A potential explanation may be that expatriates are more likely to face immigration-related stressors, as identified in a recent investigation of individuals' psychosocial and clinical profiles visiting the Emergency Department in Qatar due to suicide attempts [43]. Moreover, a review of the risk factors for suicidal behaviors highlighted the importance of social connectedness as a protective factor against lifetime suicide attempts among Filipino Americans [44]. The latter study concluded that establishing an extensive family network was a survival strategy for the Filipinos [44]. It is worth adding that most people under “other nationalities” in our sample were from the Philippines. Thus, separation from family due to immigration to Qatar might disconnect the Filipinos and other expatriates from this protective factor and predispose them to suicidal attempts. Further, a systematic review investigating suicide risk among immigrants and ethnic minorities in Europe found evidence that these two groups are at an increased risk of attempting suicide than native populations. Non-European immigrant women, including South Asian and black African origins, were at the highest risk for suicidal behaviors [45]. Apart from basic psychosocial and psychopathological risks, these individuals may be exposed to additional risk factors such as language barriers, being distant from family, and worrying about family. The review suggested that the loss of social network and status, acculturation, and less knowledge about the healthcare system may trigger suicide attempts [45].

The most common psychiatric diagnoses in the suicide group were major depressive disorder and adjustment disorder. Our results are consistent with other worldwide population studies [46] including the Arab countries [31, 38, 43] and other international studies on patients with suicide attempts [47]. Other common psychiatric diagnoses in our suicide group were substance use, psychotic disorder, bipolar disorder, and personality disorders, all of which have also been associated with an increased risk of suicide attempts [46, 48]. The most common method of attempted suicide was an overdose, previously reported in several international and regional studies [33, 49].

### Sleep patterns and suicide

Our results showed that more individuals from the suicide group reported early insomnia or difficulty falling asleep than the control group. After conducting multiple regression analyses, we found that early insomnia was independently associated with attempting suicide (Table 5). Previous studies reported mixed results regarding the most common type of insomnia associated with suicide [2]. For example, in one study with a sample from the general population, late insomnia and difficulty maintaining sleep were independent predictors of suicide attempts after controlling for demographic characteristics, psychiatric disorders, and chronic health conditions, but early insomnia was not [50]. In another study, none of the types of insomnia (hypersomnia, difficulty initiating sleep, and difficulty maintaining sleep) was an independent predictor of suicide attempts [51]. However, there is ample evidence that all three forms of insomnia (early, late, and sleep maintenance) are more prevalent in those who attempt suicide than the general population [2, 12, 51, 52].

Further, insomnia has been implicated in developing various psychiatric illnesses, which have been associated with an increased risk of suicidal thoughts and behaviors [2]. The severity of insomnia symptoms has been independently linked to suicidal ideation after controlling for depressive symptoms and anhedonia [53]. In a retrospective study, patients with depression who reported a prior suicide attempt had more sleep latency than healthy controls [54]. A 2018 nationwide retrospective cohort study in China compared patients with insomnia to those without and found that insomnia was independently associated with an increased risk for suicide attempts [55]. Using a nationally representative sample of U.S. adults, Geoffroy et al. compared those who attempted suicide with those who did not and found that early insomnia, early morning awakening, and hypersomnia were more prevalent in the suicide group. These sleep disturbances were collectively associated with an increased risk of attempting suicide independent of psychopathology [9]. It is worth noting that our sleep questionnaire covers the period before the suicidal attempt, which might indicate that insomnia can be a potential warning sign for the impending suicide attempt shown in the significant association between the two. However, this possibility cannot be confirmed in a cross-sectional study like ours. Still, other prospective and well-designed studies have shown that sleep problems can be a warning sign for further suicidal ideation and behaviors, independent of the depressive symptoms [3]. Further, a review of sleep disturbances as potential warning signs for suicide recommended the importance of screening for suicide in sleep clinics [4]. Our results also showed that most of the suicide group had no prior psychiatric history and even after the attempt, most of them were given the diagnosis of an adjustment disorder (Table 2). The association between sleep and suicide may involve several neurocognitive mechanisms. Sleep loss may lead to impaired executive functioning, risky decision making, difficulty in determining the importance of emotional stimuli, impaired emotional regulation, and increased negative affect [10]. More research and close monitoring of patients are needed to understand the role of insomnia in suicide behaviors.

According to our results, the control group had significantly more individuals who reported snoring than the suicide group. However, there was no significant difference between the two groups concerning sleep apnea. Other studies have demonstrated that depression probably mediates the association between snoring/sleep apnea and suicide, and thus they were not independent predictors of suicidal ideation [56]. Furthermore, several studies have identified an association between severe obstructive sleep apnea and depression [57–59], which increases the risk of suicidal thoughts and behaviors [60].

Our results showed that significantly more individuals from the suicide group reported a sleep quality of 2 out of 5 (higher values mean better quality), and more individuals from the control group reported a sleep quality of 5. Further, more individuals from the suicide group reported that their sleep was interrupted. Previous studies agree that poor sleep quality is associated with an increased risk of suicidal ideation and death by suicide. In one such study, including patients with major depression, poor subjective sleep quality was associated with an increased risk of suicidal ideation [61]. Another study found that non-restorative sleep and poor subjective sleep quality were significantly associated with death by suicide independent of depression [62].

We also found that more individuals in the suicide group reported sleeping more than 9 h a day. There was no significant difference in the number of individuals from the two groups who reported sleeping less than 6 h a day. In the general population and patients with depression or anxiety, 24-h sleep duration of $\ge$ 10 h and $\le$ 6 h has increased the risk of suicidal ideation and attempts across various experimental settings [51, 63, 64]. Perhaps our results did not show an association between sleeping less than 6 h and suicidal attempts due to our self-report measures. Subjects may have included the time taken to fall asleep and the time they stayed awake when their sleep was interrupted as part of the number of hours slept per night, which may have confounded the results.

Our results showed also significant gender differences in the prevalence of sleep disturbances where only males showed that all types of insomnia, short sleep duration, and poor sleep quality, were significantly more frequent in males with suicide attempt than controls. No such significant differences were seen in females. These findings are relevant in view of the known gender differences about suicidality and its attributes. Other recent studies have also reported gender differences in sleep patterns when assessing suicidal ideation and behaviors [65, 66].

### Exercise and suicide

We found that compared to the control group, individuals in the suicide group were less likely to report involvement in physical activity during the past month. Moreover, individuals in the control group were more likely to report participation in physical activity at least once per week. Simon et al. also reported similar findings; individuals with nearly lethal suicide attempts were less likely to report involvement in physical activity in the past month compared to the controls [18]. In another study with Korean adolescents, those who met the guidelines for physical activity were less likely to report a past suicide attempt [19]. A large population-based, cross-sectional study in South Korea found that those who did not report involvement in physical activity over the past year were more likely to report suicidal thoughts [63]. It is well known that acute aerobic exercise improves emotional regulation by improving the ability to attenuate negative emotions [17], enhancing resilience when dealing with life’s stressors [67]. Thus, exercise shows potential as an adjunctive treatment for people experiencing suicidal thoughts and should be investigated in future research.

### Strengths and limitations

This is the first cross-sectional study to examine the relationship between suicide and sleep/exercise patterns in the MENA region and Arab countries. It is essential to report that this study has a few limitations that should be controlled for in future studies. First, the sleep and exercise patterns were assessed using self-reported surveys, limiting the accuracy of such measures as more automated methods like actigraphy might be a better objective tool for sleep and exercise measures. However, it is worth reporting that some studies reported a moderate to strong correlation between many subjective measures and actigraphy when comparing sleep parameters in subjects with “mental ill-health” [68]. Second, this cross-sectional design limits any inferential conclusion on the relationship between sleep/exercise and suicide behaviors. Prospective studies would be more helpful to clarify the relationship between sleep/exercise and suicide. Third, the sample size was based on the duration of study and convenience sampling and not on power analysis and estimation of the differences in the sleep parameters to be analyzed, which is a better way to find significant differences. Fourth, the design of this study by including subjects with major suicidal attempts (as most of them will have a psychiatric diagnosis) does not allow to control for the fact that the mental illness can confound the significant association between sleep and suicide. Finally, some of the tools used in this study lack formal validation and consistency with other studies abroad, limiting our findings' generalizability.

## Conclusions

In conclusion, individuals experiencing insomnia symptoms should be assessed for suicidal ideation, and those with a prior suicide attempt should be monitored for insomnia symptoms. This is the first study in the Arab countries on the relationship between sleep/exercise and suicide. Our results provide a basis for further investigation of insomnia (being a warning sign for suicide) and exercise as therapeutic targets for reducing suicidality. Furthermore, sleep disturbances and exercise should be part of suicide risk assessment in all care settings to promptly prevent future suicide attempts.

## Data Availability

In order to protect the privacy and confidentiality of the research participants our de-identified data are available only upon request and after compliance with the policies and procedures of WCM-Qatar, HMC and QNRF for data sharing. Requests can be submitted to Suhaila Ghuloum at sghuloum@hamad.qa.
